# The Clinical Significance and Potential Role of C-Reactive Protein in Chronic Inflammatory and Neurodegenerative Diseases

**DOI:** 10.3389/fimmu.2018.01302

**Published:** 2018-06-07

**Authors:** Ying-yi Luan, Yong-ming Yao

**Affiliations:** ^1^Trauma Research Center, First Hospital Affiliated to the Chinese PLA General Hospital, Beijing, China; ^2^State Key Laboratory of Kidney Disease, The Chinese PLA General Hospital, Beijing, China

**Keywords:** C-reactive protein, chronic inflammation, biomarker, cardiovascular disease, diabetes

## Abstract

C-reactive protein (CRP) is an acute-phase protein synthesized by hepatocytes in response to pro-inflammatory cytokines during inflammatory/infectious processes. CRP exists in conformationally distinct forms such as the native pentameric CRP and monomeric CRP (mCRP) and may bind to distinct receptors and lipid rafts and exhibit different functional properties. It is known as a biomarker of acute inflammation, but many large-scale prospective studies demonstrate that CRP is also known to be associated with chronic inflammation. This review is focused on discussing the clinical significance of CRP in chronic inflammatory and neurodegenerative diseases, such as cardiovascular disease, type 2 diabetes mellitus, age-related macular degeneration, hemorrhagic stroke, Alzheimer’s disease, and Parkinson’s disease, including recent advances on the implication of CRP and its forms specifically on the pathogenesis of these diseases. Overall, we highlight the advances in these areas that may be translated into promising measures for the diagnosis and treatment of inflammatory diseases.

## Introduction

C-reactive protein (CRP) belongs to the family of pentraxins and exists in at least two conformationally distinct forms—such as the native pentameric CRP (pCRP) and monomeric CRP (mCRP). Studies suggest that pCRP possesses both pro-inflammatory and anti-inflammatory properties in a context-dependent manner (1). By contrast, mCRP exerts potent pro-inflammatory actions on endothelial cells, endothelial progenitor cells, leukocytes, and platelets and may amplify the inflammatory response (1). The dissociation of pCRP into pro-inflammatory mCRP might directly link CRP to inflammation. CRP is considered as a serum biomarker in patients undergoing acute inflammatory response (2–4). The elevation in baseline CRP level was shown to be useful to gauge chronic inflammation and tissue damage resulting from excessive inflammation or failure of the initial inflammatory response (5). Higher CRP concentrations may be indicative of an acute infection or inflammation and therefore are often excluded in studies of chronic inflammation. Higher CRP concentration over time, rather than spikes in CRP, may result in cardiovascular diseases (CVDs) and problems leading to atherosclerosis (6). Furthermore, some chronic inflammatory diseases such as hemorrhagic stroke, Alzheimer’s disease (AD), and Parkinson’s disease (PD) are also associated with CRP formation (7–10) (Table 1). In this review, we emphasize recent advances that may explain how conformational changes in CRP are linked to chronic inflammation and neurodegeneration.

**Table 1:** Chronic inflammatory diseases associated with CRP levels.

Disease category	Pathology/disease type	CRP (mCRP/nCRP) levels	Role and clinical significance of CRP
CVD	Atherosclerosis, chronic heart failure	Elevated mCRP levels (11)	Inflammatory biomarker, risk predictor, participant
T2DM	Insulin resistance	Elevated CRP levels (12)	Inflammatory biomarker, risk predictor, mediator
AMD	Progressive visual impairment, senile macular degeneration, blinding disease	Elevated CRP levels (13)	Inflammatory biomarker, risk predictor
Hemorrhagic Stroke	Intracerebral hemorrhage, subarachnoid hemorrhage, brain injury	Elevated CRP levels (14)	Inflammatory biomarker, risk predictor
AD	Neurodegenerative disorder, dementia	Reduced/elevated CRP levels (15)	Inflammatory biomarker, no causal role
PD	Neurodegenerative disorder, motor symptoms	Elevated CRP levels (16)	Inflammatory biomarker, risk predictor

*CRP plays an important role in the progression of various chronic inflammatory diseases. This table lists diseases associated with nCRP and monomeric CRP (mCRP) levels. Although any potential mechanism underlying the effect of CRP on these processes is incompletely elucidated, its clinical significance appears to be positive*.

*CVD, cardiovascular disease; T2DM, type 2 diabetes mellitus; AD, Alzheimer’s disease; PD, Parkinson’s disease; AMD, age-related macular degeneration; CRP, C-reactive protein; nCRP, native CRP*.

## CRP and CVDs

Elevated levels of numerous inflammatory biomarkers have been implicated to predict adverse cardiovascular events. Several studies indicate the predictive CRP values in patients with acute coronary syndrome (ACS). The routine biomarkers of CVD include troponin I (cTnI) and creatine kinase isoenzyme (CK-MB) that are mainly synthesized in cardiac muscle cells. Both cTnI and CK-MB may be detected in the blood during severe ischemia, degeneration, and necrosis of cardiomyocytes; but lack the sensitivity to detect minute damage to cardiomyocytes (17, 18). CRP is the biomarker that most strongly correlates with future cardiovascular events and may be slightly elevated during the early stage of myocardial vascular inflammation (7, 19). Unlike other markers of inflammation, CRP levels are stable over long periods and display no diurnal variation. In addition, these may be inexpensively measured with available high-sensitivity assays and have shown specificity in terms of the prediction of CVD risk (20). At present, high-sensitivity CRP (hs-CRP) is gaining popularity under clinical settings, as it allows the detection of lower levels of CRP. A moderate increase in hs-CRP is thought to predict an increased risk of coronary events in unstable angina pectoris patients and its detection is a reliable predictor of the risk of CVDs (21–23). During the course of atherosclerotic plaque formation, the stimulation of the local inflammatory response may lead to elevated levels of hs-CRP, which has been identified as a risk factor for atherosclerosis (24). These findings have highlighted the significant importance of hs-CRP for the evaluation of atherosclerotic inflammation. However, a recent meta-analysis called into question the clinical value of CRP as a predictor of CVD risk. In their prospective study, Danesh et al. (25) revealed CRP as a relatively modest predictor of CVD. Many studies have identified elevated serum CRP levels in response to cardiovascular events and that CRP levels may strongly and independently predict adverse cardiovascular events, including myocardial infarction (MI), ischemic stroke, and sudden cardiac death (26).

Although CRP is an inflammatory biomarker, its involvement in the pathogenesis of CVD is questionable. CRP has been demonstrated to exhibit prothrombotic property, and high concentrations of CRP may activate the coagulation system, resulting in an increase in the level of prothrombin and D-dimer (27, 28). In a clinical study with a long follow-up, the investigators reported high predictive values of CRP in atherosclerotic thrombotic events after the implantation of drug-eluting stents (29). Studies investigating the relationship between stent thrombosis, hs-CRP levels, and statin therapy revealed that statins may only reduce the formation of early stent thrombosis in patients with high levels of hs-CRP. Rosuvastatin was similarly shown to markedly reduce the incidence of CVD and CVD-associated mortality and decrease levels of CRP and low-density lipoprotein (LDL) (30). Furthermore, Eisenhardt et al. (31) found a localized dissociation mechanism of pCRP into mCRP mediated by activated platelets, thereby resulting in the deposition of mCRP in atherosclerotic plaques. A clinical trial has demonstrated the effectiveness of statins in reducing the incidence of future cardiovascular events is associated with decreased pCRP level (32). Ruptured plaques and inflamed tissues in patients with ACS were shown to be more prone to opsonization by mCRP, leading to the consumption of autoantibodies against mCRP (33). In addition, multiple epidemiological studies have revealed the participation of CRP in the pathogenesis of CVDs based on genetic polymorphisms that affect CRP levels (34). However, some epidemiological studies failed to support the notion that the common variation in CRP gene had an alternative effect on the occurrence of coronary heart diseases. A Mendelian randomization study of over 28,000 cases and 100,000 controls found that a lack of concordance between the effect of the CRP genotypes on the risk of coronary heart diseases and CRP levels argued against a causal association of CRP with coronary heart disease (28). Using genetic variants as the unconfounded proxies of CRP concentration, Mendelian randomization meta-analysis of individual participant data from 47 epidemiological studies showed that CRP concentration itself was unlikely to be the modest causal factor in coronary heart disease (35). Steady state CRP levels in serum are influenced by CRP gene haplotypes. Although elevated CRP level has lately been found to be a consistent and relatively strong risk factor for CVD, no association was observed between CRP gene haplotypes and coronary heart disease (36). A prospective, nested case-control study design from the Physicians’ Health Study cohort demonstrated that none of the single nucleotide polymorphisms related to higher CRP levels showed any association with the risk of incident MI or ischemic stroke (37). Therefore, these epidemiological data indicate a significant interaction between both genetic and environmental factors and increased CRP levels that predict a greater risk of CVD events.

**Figure 1:**
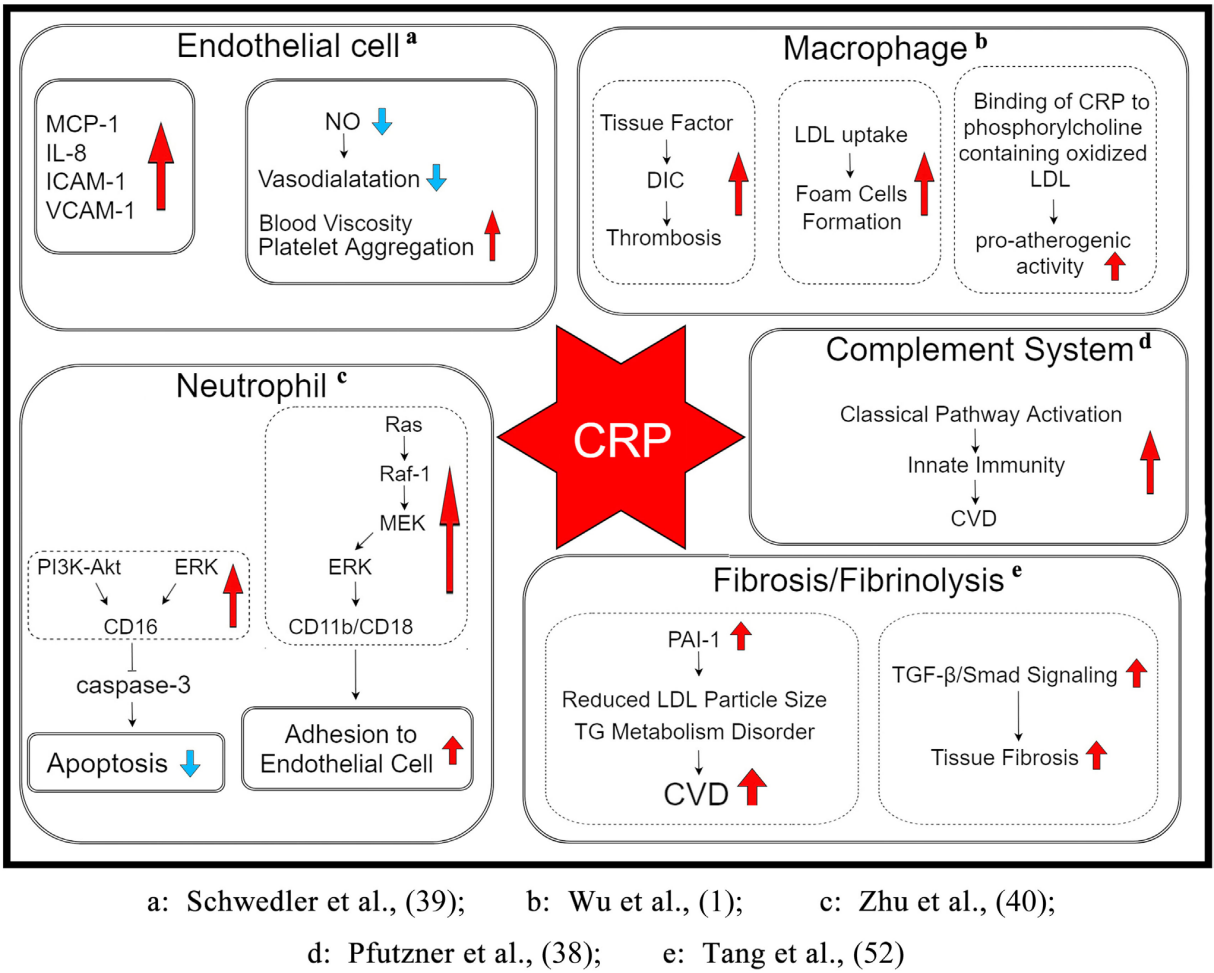
The potential mechanism underlying the role of CRP in the pathogenesis of CVDs. CRP may be involved in various stages through its direct influence on pathophysiological processes such as the activation of endothelial cells and macrophages, inhibition of apoptosis of neutrophils and expression of endothelial NO synthase, stimulation of the complement cascade, enhancement of PAI-1 activity and LDL uptake, accumulation of lipid and thrombosis, and upregulation of pro-inflammatory cytokine expression. Abbreviations: DIC, disseminated intravascular coagulation; LDL, low-density lipoprotein; CVD, cardiovascular diseases; TG, triglyceride; MCP-1, monocyte chemoattractant protein 1; ICAM-1, intercellular adhesion molecule 1; VCAM-1, vascular cell adhesion molecule 1; NO, nitric oxide; PI3K, phosphatidylinositol 3-kinase; ERK, extracellular signal-regulated kinase; MAPK, mitogen-activated protein kinase; PAI-1, plasminogen activator inhibitor-1; TGF-β, transforming growth factor-β; CRP, C-reactive protein.

Some basic research shows that the inflammatory response plays a central role in various phases of atherosclerosis, i.e., from the initial recruitment of circulating leukocytes to the arterial wall to the rupture of unstable plaques, thereby resulting in the clinical manifestations of the disease. CRP may be critically involved in each of these stages by directly influencing processes, including complement activation, apoptosis, endothelial nitric oxide (NO) synthase inhibition, vascular cell activation, monocyte recruitment, lipid accumulation and thrombosis, and pro-inflammatory cytokine formation (38). Taken together, CRP appears to play a pivotal role in many aspects of CVD, as described below (Figure 1):

- mCRP induces endothelial cells to produce monocyte chemoattractant protein 1, interleukin-8 (IL-8), intercellular adhesion molecule 1, and vascular cell adhesion molecule 1 (39).
- mCRP inhibits the apoptosis of neutrophils that is partly meditated by the activation of the low-affinity immunoglobulin G immune complex receptor FcγRIII (CD16) *via* stimulation of phosphatidylinositol 3-kinase (PI3K)/protein kinase B and extracellular signal-regulated kinase (ERK)/mitogen-activated protein kinase (MAPK)-ERK (MEK) signaling pathways, leading to the inhibition of caspase-3. This process is partly mediated by the activation of neutrophil ERK *via* the Ras/Raf-1/MEK cascade that increases CD11b/CD18 expression, thereby promoting adhesion to endothelial cells (40).
- C-reactive protein activates macrophages to secrete tissue factor, a powerful procoagulant, which may lead to disseminated intravascular coagulation and thrombosis during inflammatory states.
- C-reactive protein increases the uptake of LDL into macrophages and enhances the ability of macrophages to form foam cells. It binds to phosphocholine of oxidized LDL.
- Through the above action, CRP induces the classical complement pathway and directly activates and amplifies the innate immunity, a process that has already been associated with the initiation and progression of CVD.
- C-reactive protein inhibits endothelial NO synthase expression. NO, an important signaling molecule, is closely associated with the regulation of vasodilatation, blood rheology, platelet aggregation, and other physiological as well as pathological processes.
- C-reactive protein upregulates plasminogen activator inhibitor-1 (PAI-1) expression and activity. PAI-1 reduces LDL particle size in conjunction with triglyceride metabolism disorder, leading to an increased risk of CVD (41).
- C-reactive protein mediates tissue fibrosis in CVD by activating transforming growth factor-β (TGF-β)/Smad signaling *via* TGF-β1-dependent and -independent mechanisms.

## CRP and Type 2 Diabetes Mellitus (T2DM)

Type 2 diabetes mellitus is a serious disease associated with high morbidity and mortality. CRP, tumor necrosis factor-alpha, and IL-6 may be triggered by the excessive adipose tissue to activate insulin signaling pathways, resulting in insulin resistance that eventually progresses into T2DM (42). Cross-sectional and prospective studies have demonstrated a relationship between elevated CRP levels and increased risk for T2DM (43). Higher levels of hemoglobin A1c (HbA1c, %), the indicator of overall glycemic control in diabetics, were positively correlated with elevated CRP levels even after adjustment, as seen in elderly patients with T2DM (44, 45). In comparison to subjects with normal fasting glucose, patients with diabetes and impaired fasting glucose level (defined by the reduction in glucose from 110 to 100 mg/dL and/or elevated plasma glucose level at 2 h in the oral glucose tolerance test) showed a strong increase in hs-CRP (46, 47). Furthermore, clinical studies showed elevated serum concentrations of fetuin-A, vascular endothelial growth factor, and CRP in T2DM patients with diabetic retinopathy (DR), suggestive of the increased risk of DR with high CRP level (48).

Based on the multitude of clinical observations, CRP appears to be not only a marker of chronic inflammation but also a mediator of kidney diseases in basic research. CRP is known to bind to its receptor FcγRII (CD32/CD64) and directly activate or interact with a number of signaling pathways in the process of inflammation, fibrosis, and aging. CRP may activate the mechanistic target of rapamycin (mTOR) signaling under diabetic conditions (49). To date, the most consistent animal data in CRP research were obtained with the diabetic mouse model of CRP; CRP was shown to clearly enhance renal fibrosis in diabetic nephropathy (T2DN) *via* CD32b-Smad3-mTOR signaling (50). In addition, CRP could activate Smad3 *via* both TGF-β-dependent and ERK/MAPK crosstalk mechanisms, leading to the direct binding of Smad3 to p27. The suppression of Smad3 or FcγRII CD32 may result in the inhibition of CRP-induced p27-dependent G1 cell cycle arrest and promotion of CDK2/cyclin E-dependent G1/S transition of tubular epithelial cells (51). Moreover, CRP could markedly mediate tissue fibrosis in several cardiovascular and kidney diseases by activating the TGF-β/Smad3 pathway (52).

## CRP and Age-Related Macular Degeneration (AMD)

Age-related macular degeneration, an acquired disease of the macula, is characterized by progressive visual impairment, owing to the late-onset neurodegeneration of the photoreceptor-retinal pigment epithelial complex (53–57). Chronic inflammation is thought to be critically involved in the pathophysiology of AMD. A cross-sectional study documented an obviously higher CRP level in the exudative form of AMD (eAMD) as compared to that observed in the early form (56). Higher CRP levels were closely associated with the higher risk of exudative AMD (55, 58–60). In comparison with pCRP, pro-inflammatory mCRP strongly influenced endothelial cell phenotypes, indicative of its potential role in choroidal vascular dysfunction in AMD (61). A recent observational study of elderly European patients by Cipriani et al. (62) suggested no causal association between CRP concentrations and AMD. However, complement activation may be involved in the development of AMD. It was found that mCRP played a role in choroidal vascular dysfunction in AMD by influencing endothelial cell phenotypes *in vitro* and *ex vivo* (63). In addition, high levels of CRP could activate the complement system at the retina/choroid interface and contribute to chronic inflammation and subsequent tissue damage (64). These clinical results indicate that CRP plays an important role in the pathogenesis of AMD and may be used to assess the severity of AMD. Plasma levels of CRP are independently associated with the risk of AMD, but whether CRP is causally associated with AMD or acts as a mere marker of AMD is uncertain.

## CRP and Hemorrhagic Stroke

During the initial stages of hemorrhagic stroke, including intracerebral hemorrhage and subarachnoid hemorrhage, the reflex mechanisms are activated to protect cerebral perfusion. The inflammatory process and hyperglycemia are involved in the spontaneous intracerebral hemorrhage (sICH) as well as the progression of sICH-induced brain injury (65, 66). Several prospective studies have reported the association between higher CRP levels and increased disability risk of ischemic stroke (67). CRP elevation displays negative prognostic implications for many conditions, while elevations in CRP as a consequence of the major acute-phase response following ischemic or hemorrhagic stroke are associated with death and vascular complications (68). In a cross-sectional study, an increase in hs-CRP level was observed in patients with ischemic infarction but not in those with hemorrhagic stroke, suggestive of the role of hs-CRP in the initial diagnosis of the stroke type (69). Moreover, CRP was an independent predictor of mortality and its expression was significantly correlated with poor clinical outcomes in sICH (70–72). Although several studies have shown the higher level of CRP in patients with ischemic stroke, its potential role in various stroke types, particularly in ischemic and hemorrhagic stroke, needs to be investigated.

## CRP and AD

Alzheimer’s disease is a neurodegenerative disorder characterized by gradually progressive cognitive decline and functional impairment. Neuroinflammation may play a potential role in AD pathogenesis (73, 74). However, the precise mechanism related to AD phenotype remains unclear. CRP was clearly recognized in the senile plaques (SP) from patients with AD using immunostaining, implying that the process of SP formation may include an acute-phase inflammatory state and/or the formation of CRP (75). A meta-analysis included 10 cross-sectional studies and showed no significant difference in the serum CRP level between AD patients and normal controls, whereas patients with mild and moderate AD had lower serum CRP levels as compared with the healthy controls by Mini-Mental State Examination scores (15), indicating that the diagnostic value of CRP for mild and moderate AD may be useful in clinical practice. Most studies support the reduced plasma CRP levels in mild and moderate AD patients and indicate its potential role as a representative systemic inflammatory marker for the diagnosis of AD. In addition, lower CRP levels are associated with more rapid cognitive and functional decline (76). The elevated CRP level was associated with an increased risk of AD (77), while such elevation appeared to diminish and fall below the level observed in nondemented controls after the clinical manifestation of the disease. In addition, the influence of CRP and homocysteine (Hcy) on patients suffering from AD was assessed and both CRP and Hcy were found to play no role in the development of AD (78). Strang et al. (79) recently demonstrated the association between mCRP in AD patients and beta-amyloid (A-β) plaques, which may induce the dissociation of pCRP into individual monomers. Moreover, basic research of human AD/stroke patients revealed that high mCRP levels from infarcted core regions were associated with the reduced expression of A-β/Tau, suggestive of the role of mCRP in promoting dementia after ischemia (80). Taken together, a direct functional effect elicited by CRP may, at least in part, explain the pathogenesis of AD.

## CRP and PD

Parkinson’s disease is a neurodegenerative disorder pathologically characterized by dopaminergic neuronal death and the presence of Lewy bodies (81). Previous studies have highlighted the key role of neuroinflammatory reactions in the pathogenesis of PD and patients with PD were shown to exhibit higher levels of serum hs-CRP. Patients with more severe PD, classified according to the Hoehn–Yahr staging system, had significantly higher levels of hs-CRP than those at an earlier stage and non-Parkinson’s control subjects (82). A cross-sectional study suggested that CRP may play an important role in the development of PD and elevations in the plasma CRP level correlated with an increased risk of PD. Baseline CRP concentrations were recently shown to be associated with the risk of death and predicted life prognosis of patients with PD (81). A retrospective analysis further supported the association between baseline plasma CRP levels and motor deterioration and predicted motor prognosis in patients with PD; these associations were independent of sex, age, PD severity, dementia, and use of antiparkinsonian agents (83). Although formal demonstration of the mechanism of action of CRP in the pathogenesis of PD is currently lacking, there is a continuous increase in the experimental data, which is in line with the aforementioned concept.

## Conclusion

As a nonspecific marker of inflammation, CRP plays a vital role in the monitoring of bacterial infection, inflammation, neurodegeneration, tissue injury, and recovery. Chronic inflammation may be a continuation of an acute or a prolonged low-grade form, which is increasingly recognized as an important issue with social and economic implications. CRP levels are observed to be increased during acute-phase inflammation as well as chronic inflammatory diseases. From both experimental and clinical data, increasing evidence suggest that elevated CRP concentrations are associated with an increased risk of CVD, T2DM, AD, hemorrhagic stroke, PD, and AMD. Moreover, CRP is not only an excellent biomarker of chronic inflammation but also acts as a direct participant in the pathological process (84). The differentiation between the physiological and pathophysiological CRP levels may allow better management of inflammation-related diseases. Although the clinical significance and underlying mechanisms of CRP in chronic inflammatory and neurodegenerative diseases are incompletely elucidated, further research is required in order to differentially characterize the roles of CRP isoforms (pCRP, facilitator, versus mCRP, effector) in chronic inflammation onset and progression. A better understanding of CRP activation and dissociation is essential to develop therapeutic strategies to minimize tissue injury, which may further improve the outcome of chronic inflammatory diseases.

## Author Contributions

We affirm that all authors have contributed to, seen, and approved the final, submitted version of the manuscript and are willing to convey copy right to Frontiers in Immunology.

## Conflict of Interest Statement

The authors declare that the research was conducted in the absence of any commercial or financial relationships that could be construed as a potential conflict of interest. The reviewer JJA and handling Editor declared their shared affiliation.
